# A New Work on Dental Medicine

**Published:** 1883-12

**Authors:** 


DENTAL MEDICINE.—Messrs. P. Blackiston, Son & Co., Philadelphia, the widely known publishers of Medical and Dental works will issue during the present month, December, 1883, a work prepared especially for dental students, which will embrace a complete dental Materia Medica and Therapeutics describing such medicinal agents as are employed in dental practice, and relating to the source or derivation, the medical properties and action, the therapeutic uses, the different combinations, the doses, and the *dental uses and modes of application* of such dental remedies, together with valuable formulæ, arrayed under each subject for the treatment of different pathological conditions. The object of the author, Ferdinand J. S. Gorgas, A. M., M. D., D. D, S., has been to make this work a practical and useful treatise on dental medicine.

